# Supplementary material for: Obeticholic Acid Derivative, T-2054 Suppresses Osteoarthritis via Inhibiting NF-κB-Signaling Pathway
Source: Int J Mol Sci. 2021 Apr 7;22(8):3807. doi: 10.3390/ijms22083807 (PMC8067620; doi:10.3390/ijms22083807)
Supplement: Supplementary file 1 [file ijms-22-03807-s001.zip › Supplement/Supplement figures.docx]

**Supplement figures**

**Characterization of compounds**

^1^H (400 MHz or 500 MHz) and ^13^C (100 MHz or 125 MHz) NMR spectra were recorded on Bruker 400 MHz or 500 MHz spectrometer with CDCl_3_, CD_3_OD or DMSO-*d*_6_ as solvent and tetramethylsilane (TMS) as the internal standard. All chemical shift values were reported in units of δ (ppm). The following abbreviations were used to indicate the peak multiplicity: s = singlet; d = doublet; t = triplet; m = multiplet. High-resolution mass data were obtained on a Bruker micrOTOF-Q II spectrometer.

**Characterization of compounds**

Compound **T-2035**:

Characterization: ^1^H NMR (500 MHz, DMSO-*d*6) *δ* 12.00 (s, 1H), 7.14 (s, 1H), 4.24 (d, *J* = 5.3 Hz, 1H), 3.60 (s, 1H), 1.01 (s, 3H), 0.90 (t, *J* = 7.3 Hz, 3H), 0.84 (d, *J* = 6.5 Hz, 3H), 0.61 (s, 3H). ^13^C NMR (125 MHz, CDCl_3_) *δ* 174.85, 141.59, 131.70, 111.15, 68.73, 55.43, 50.08, 43.15, 42.04, 41.01, 39.94, 39.78, 36.63, 34.89, 32.90, 31.59, 30.74, 30.68, 27.79, 23.20, 23.09, 22.10, 21.09, 20.01, 18.13, 11.78, 11.75. HRMS (ESI) m/z: calcd for C_27_H_43_N_2_O_3_ [M+H]^+^, 443.3268, found 443.3239.

Compound **T-2036**

Characterization: ^1^H NMR (500 MHz, DMSO-*d*6) *δ* 11.92 (s, 1H), 8.27 (s, 1H), 4.39 (d, *J* = 5.4 Hz, 1H), 3.61 (s, 1H), 1.04 (s, 3H), 0.90 (t, *J* = 7.3 Hz, 3H), 0.84 (d, *J* = 6.5 Hz, 3H), 0.61 (s, 3H). HRMS (ESI) m/z: calcd for C_27_H_41_NNaO_4_ [M+Na]^+^, 466.2928, found 466.2932.

Compound **T-2055-2**

Characterization: ^1^H NMR (500 MHz, CD_3_OD) *δ* 3.76 (s, 1H), 3.25 (d, *J* = 16.9 Hz, 1H), 1.13 (s, 3H), 0.99 (t, *J* = 7.2 Hz, 3H), 0.92 (d, *J* = 6.5 Hz, 3H), 0.71 (s, 3H). HRMS (ESI) *m/z*: calcd for C_28_H_42_N_2_NaO_5_ [M+Na]^+^, 509.2986, found 509.2989.

Compound **T-2056**

Characterization: ^1^H NMR (500 MHz, CD_3_OD) *δ* 3.76 (s, 1H), 3.11 (d, *J* = 16.8 Hz, 1H), 1.15 (s, 3H), 0.99 (t, *J* = 7.3 Hz, 3H), 0.93 (d, *J* = 6.5 Hz, 3H), 0.71 (s, 3H). HRMS (ESI) *m/z*: calcd for C_28_H_41_NNaO_6_ [M+Na]^+^, 510.2826, found 510.2833.

Compound **T-2054**

Characterization: ^1^H NMR (500 MHz, DMSO-*d*_6_) *δ* 11.98 (s, 1H), 8.26 (s, 1H), 4.31 (d, *J* = 5.2 Hz, 1H), 3.60 (s, 1H), 2.48 (s, 3H), 1.05 (s, 3H), 0.91 (t, *J* = 7.0 Hz, 3H), 0.83 (d, *J* = 6.5 Hz, 3H), 0.61 (s, 3H).^13^C NMR (125 MHz, DMSO- *d_6_*) *δ* 167.40, 164.67, 155.86, 124.75, 99.98, 69.07, 55.85, 50.31, 42.84, 42.46, 41.22, 40.24, 40.08, 37.14, 35.51, 35.31, 33.94, 31.28, 31.18, 30.72, 28.19, 25.69, 23.45, 22.80, 22.52, 21.03, 18.58, 12.19, 12.17. HRMS (ESI) *m/z*: calcd for C_29_H_45_N_2_O_3_ [M+H]^+^, 469.3425, found 469.3406.

Compound **T-2052**

Characterization: ^1^H NMR (500 MHz, DMSO-*d*_6_) *δ* 11.94 (s, 1H), 7.83 (s, 1H), 6.08 (s, 2H), 4.25 (d, *J* = 5.2 Hz, 1H), 3.59 (s, 1H), 1.01 (s, 3H), 0.92–0.88 (m, 3H), 0.84 (d, *J* = 6.5 Hz, 3H), 0.61 (s, 3H).^13^C NMR (125 MHz, DMSO-*d_6_*) *δ* 175.32, 167.88, 162.56, 156.70, 115.97, 69.08, 55.85, 50.34, 43.07, 42.50, 41.31, 40.24, 40.08, 37.02, 35.72, 35.31, 33.74, 31.19, 31.14, 30.68, 28.21, 23.48, 22.89, 22.55, 21.03, 18.59, 12.22, 12.18. HRMS (ESI) *m/z*: calcd for C_28_H_44_N_3_O_3_ [M+H]^+^, 470.3377, found 470.3376.

Compound **T-2005**

Characterization: ^1^H NMR (500 MHz, DMSO-*d*_6_) *δ* 11.91 (s, 1H), 10.73 (s, 1H), 7.64 (d, *J* = 7.5 Hz, 1H), 7.19 (d, *J* = 7.9 Hz, 1H), 6.93 (t, *J* = 7.4 Hz, 1H), 6.89–6.80 (m, 1H), 3.64 (s, 1H), 1.12–0.99 (m, 6H), 0.88 (d, *J* = 6.5 Hz, 3H), 0.66 (s, 3H). ^13^C NMR (125 MHz, CD_3_OD) *δ* 178.09, 138.41, 138.05, 128.31, 122.31, 121.50, 119.37, 111.63, 110.86, 73.17, 61.50, 57.19, 51.95, 45.86, 43.76, 42.52, 41.14, 38.49, 36.66, 36.04, 32.26, 32.00, 29.18, 24.42, 24.01, 23.23, 21.25, 20.87, 18.80, 14.47, 14.18, 12.43. HRMS (ESI) *m/z*: calcd for C_32_H_45_NNaO_3_ [M+Na]^+^, 514.3292, found 514.3288.

Compound **T-2055-1**

Characterization:^1^H NMR (500 MHz, CDCl_3_) *δ* 4.12 (q, *J* = 7.1 Hz, 2H), 3.77 (s, 1H), 1.25 (s, 3H), 0.99 (s, 3H), 0.94 (d, *J* = 6.5 Hz, 3H), 0.89 (t, *J* = 7.4 Hz, 3H), 0.69 (s, 3H). ^13^C NMR (125 MHz, DMSO-*d_6_*) *δ* 175.37, 162.54, 130.08, 116.08, 104.36, 69.09, 60.01, 55.88, 50.51, 42.91, 42.46, 41.27, 40.45, 40.41, 40.24, 40.08, 36.81, 35.34, 33.61, 32.48, 31.25, 31.16, 28.24, 23.51, 22.52, 21.58, 18.55, 14.73, 12.21, 12.18. HRMS (ESI) *m/z*: calcd for C_30_H_46_N_2_NaO_5_ [M+Na]^+^, 537.3304, found 537.3322.
