# Supplementary material for: Obeticholic Acid Derivative, T-2054 Suppresses Osteoarthritis via Inhibiting NF-κB-Signaling Pathway
Source: Int J Mol Sci. 2021 Apr 7;22(8):3807. doi: 10.3390/ijms22083807 (PMC8067620; doi:10.3390/ijms22083807)
Supplement: Supplementary file 1 [file ijms-22-03807-s001.zip › Supplement/Table S1.docx]

**Table S1 Primer sequence**

| **Primer** | **Sequence (5’-3’)** |
| --- | --- |
| IL-6- F | TTCGGTCCAGTTGCCTTCTC |
| IL-6- R | TCTTCTCCTGGGGGTACTGG |
| IL-8-F | TCTGTCTGGACCCCAAGGAA |
| IL-8- R | ATGAATTCTCAGCCCTCTTCAA |
| TNF-α-F | GCTGCACTTTGGAGTGATCG |
| TNF-α-R | CTTGTCACTCGGGGTTCGAG |
| SOX9-F | GTGCAAGCTGGCAAAGTTGA |
| SOX9-R | TGCTCAGTTCACCGATGTCC |
| MMP9-F | GTACTCGACCTGTACCAGCG |
| MMP9-R | AGAAGCCCCACTTCTTGTCG |
| ADAMTS5-F | AAGAGGAGGAGGAGGAGGAGGAG |
| ADAMTS5-R | AATGGTTGTGAGCTGCCGTATGG |
| Actin-F | GTACGCCAACACAGTGCTG |
| Actin-R | CGTCATACTCCTGCTTGCTG |
